# Experimental Treatment of Mucinous Peritoneal Metastases Using Patient-Derived Xenograft Models

**DOI:** 10.1016/j.tranon.2020.100793

**Published:** 2020-05-21

**Authors:** Karianne Giller Fleten, Christin Lund-Andersen, Stein Waagene, Torveig Weum Abrahamsen, Yrr Mørch, Kjetil Boye, Annette Torgunrud, Kjersti Flatmark

**Affiliations:** a Department of Tumor Biology, Institute for Cancer Research, Oslo University Hospital, The Norwegian Radium Hospital, Oslo, Norway; b Faculty of Medicine, Institute of Clinical Medicine, University of Oslo, Oslo, Norway; c Department of Biotechnology and Nanomedicine, SINTEF, AS, Trondheim, Norway; d Department of Oncology, Oslo University Hospital, Oslo, Norway

## Abstract

Mucinous peritoneal metastases (PM) generally respond poorly to systemic treatment, and there is a clear unmet need for new treatment strategies to improve survival and quality of life for patients with PM. In this work, the growth inhibitory effect of five drugs (oxaliplatin (OXA; 5 mg/kg), irinotecan (IRI; 60 mg/kg), cabazitaxel (CBZ; 15 or 30 mg/kg), regorafenib (REG; 10, 30 or 60 mg/kg), and capecitabine (CAP; 359 or 755 mg/kg) was investigated in three orthotopic patient-derived xenograft models that mimic mucinous PM.

Drugs were administered intraperitoneally (i.p.) as monotherapy weekly for 4 weeks (OXA, IRI), as one single i.p. injection (CBZ), or orally (REG, CAP) daily 5 of 7 days per week for four weeks, and i.p. tumor growth and survival were monitored and compared between treatment groups. The i.p. administered drugs (OXA, IRI, CBZ) had the strongest growth inhibitory effect, with OXA being most efficacious, completely inhibiting tumor growth in the majority of the animals. CBZ and IRI also strongly inhibited tumor growth, but with more variation in efficacy between the models. A moderate reduction in tumor growth was observed in all models treated with REG, while CAP had little to no growth inhibitory effect. Targeted next-generation-sequencing has identified mutational profiles typically associated with PM (mutations in *KRAS*, *GNAS*, and *BRAF* oncogenes), supporting the representativeness of the models. The results presented in this work support the continued exploration of i.p. treatment protocols for PM, with OXA remaining and CBZ emerging as particularly interesting candidates for further studies.

## Introduction

Peritoneal metastases represent an important therapeutic challenge, since patients generally respond poorly to systemic chemotherapy and targeted treatments [1]. A possible explanation could be that cancers with unfavorable molecular subtypes tend to metastasize to the peritoneal surface. Indeed, colorectal cancers (CRC) with mucinous and signet ring cell differentiation often give rise to peritoneal metastases (PM), and these tumors exhibit poor responses to several of the commonly used chemotherapeutic drugs [2,3]. Another strictly peritoneal mucinous entity is pseudomyxoma peritonei (PMP), which is a rare cancer commonly arising from mucinous tumors of the appendix, and which is also similarly poorly responsive to systemic chemotherapy [4,5]. Locoregional treatment involving cytoreductive surgery (CRS) and intraperitoneal chemotherapy represents standard-of-care in resectable cases, but when locoregional treatment fails and for patients who are not eligible for such treatment, new treatment options are needed [[6], [7], [8], [9]].

We have generated orthotopic patient-derived xenograft models that mimic mucinous PM, and that were shown to closely resemble the original disease with respect to growth pattern and protein expression [10,11]. We previously investigated single intraperitoneal (i.p.) injections of two drugs that are commonly used as components of hyperthermic intraperitoneal chemotherapy (HIPEC), mitomycin C (MMC) and oxaliplatin (OXA), identifying MMC as the more efficacious drug compared to OXA when administered as a single i.p. injection [12]. In this study, we investigated drugs that are part of standard systemic chemotherapy in CRC; OXA, irinotecan (IRI) and capecitabine (CAP); and included two less commonly used drugs in this setting, cabazitaxel (CBZ) and regorafenib (REG).

## Materials and Methods

### *In Vivo* Experiments

All procedures and experiments involving animals were approved by the Norwegian Food Safety Authority (application ID #11836, #11946, and #18209), and were conducted according to the recommendations of the European Laboratory Animals Science Association. Female athymic foxn 1^nu^ were bred at Department of Comparative Medicine, Oslo University Hospital, and kept in a specific pathogen-free environment at constant temperature (22 ± 1 °C) and humidity (62 ± 5%), 15 air changes/hour and a 12-hour light/dark cycle. Food and water were supplied ad libitum*,* and the mice were given paper and card board houses for environmental stimulation. A maximum of 10 mice were housed in each cage. The model establishment was previously described [10,11], and the models PMP-2, PMCA-1 and PMCA-3 were used in these experiments. All models were established by implanting peritoneal tissue samples collected at the time of CRS-HIPEC. PMP-2 and PMCA-3 were derived from patients with appendiceal primaries, whereas the PMCA-1 patient had a primary rectal carcinoma. PMP-2 was classified as peritoneal mucinous carcinomatosis intermediate histological type (PMCA-I) based on the Ronnett classification [13], while PMCA-3 was a high grade PMP with signet ring cell differentiation. For treatment experiments 125–200 μl mucinous ascites was injected (i.p), and treatments were initiated the following day to simulate the clinical situation after CRS with a very low remaining tumor load intraabdominally. Mice were randomly assigned to treatment groups of 6 mice. The mice were routinely examined by experienced animal technicians, and sacrificed when abdominal distension was clearly visible, at which time approximately 4–5 g of mucinous tumor tissue would be weighed at autopsy. Occasionally, the tumor had a more solid growth pattern with a necrotic core, and in these cases the mice developed cachexia, necessitating sacrifice. Animals with no sign of tumor growth were sacrificed 100 days (range 100–103 days) after experiment initiation, which in all experiments was at least twice the median time of the survival of the vehicle treated animals. Tumor growth/response was quantified by calculating a growth index [12], combining the key parameters survival time (in days) and tumor load at the time of sacrifice (in g), using the equation:

$$\text{Growth index}=\text{tumor weight}+\left(\left(T_{\text{total}}-T_{A}\right)/T_{\text{Total}}\right)\;x\;10.$$

where T_A_ is the time from start of the experiment until sacrifice of the animal, and T_Total_ is the total duration of the experiments.

Of the 184 animals included in the experiments, seven were excluded from analysis for the following reasons: wrong treatment given (n = 1) (PMP-2), mucin not weighed (n = 5) (PMCA-3), development of ascites with no visible tumor growth (n = 1) (PMP-2).

### Drugs

OXA (Fresenius Kabi, Germany) was diluted in 5% glucose; i.p. injections of 5 mg/kg were administrated weekly for 4 weeks. CBZ (TXD-258, BioChemPartner Co. Ltd., Shanghai, China) was dissolved in polysorbate 80 (40 mg/ml), diluted in 13% ethanol to a working concentration of 10 mg/ml, and further diluted in 0.9% NaCl; i.p. injections of 15 and 30 mg/kg were administrated once. IRI (Pfizer, New York, NY, USA), (prodrug that is metabolized to SN-38 [14]) was diluted in 0.9% saline; i.p. injections of 60 mg/kg were administrated weekly for 4 weeks. Injection volumes were in the range of 200–250 μl, according to body weight (10 μl/g mouse). REG (kindly donated by Bayer) was dissolved in DMSO, further diluted in PEG400, and administrated by oral gavage 5 of 7 days per week for 4 weeks. Tablets of CAP (Roche, Hertfordshire, UK) (prodrug that is metabolized to 5-fluorouracil [15]) were suspended in a vehicle consisting of 50 mM citrate buffer and 4.67% (w/v) arabic gum (pH 6.0), and 359 or 755 mg/kg was administrated by oral gavage 5 of 7 days per week for 4 weeks. Vehicle treated animals received DMSO/PEG400 5 of 7 days per week for 4 weeks corresponding to regorafenib treatment. All of the drugs and doses used in the experiments were well tolerated, and no toxicity, defined as a weight reduction of more than 15%, was observed.

Drug regimens were chosen based on the maximum tolerable doses of the drugs previously published or based on in-house experience. For CAP, 359 mg/kg was chosen based on previous experience of efficacy in another model [16], but as this dose had limited effect, experiments were also performed with the defined maximum tolerable dose of 755 mg/kg [17]. The treatment regimens were chosen based on the recommended administration route and frequency for each drug.

### DNA Sequencing

Tumor tissue samples from PDX models were homogenized and disrupted using TissueLyzer LT from QIAGEN. DNA was then extracted from the lysate using the NucleoSpin Tissue kit (Macherey Nagel, Düren, Germany). DNA concentrations and purity were evaluated using ThermoFisher NanoDrop spectrophotometer, and the Abs_260/280_> 1.8 for all the samples. Targeted DNA sequencing was performed using the Ion Torrent PGM Personal Genome Machine and the Ion AmpliSeqTM Cancer Hotspot Panel v2 (Thermo Fisher Scientific, Waltham, MA, USA), covering ~2800 hotspot mutations in 50 cancer related genes. The Torrent Suite Variant Caller, with the manufacturer's recommended settings, was used to generate single nucleotide variants and small insertions/deletions with a variant allele frequency threshold of two percent. The sequencing depth exceeded 500× for 98% of all amplicons (median depth of 4376×). Every detected mutation was manually reassessed using Integrative Genomics Viewer and functionally annotated with ANNOVAR [18], using RefSeq as the underlying gene model and information from the 1000 Genomes Project (1000genomes.org) and the Catalogue of Somatic Mutations in Cancer (cancer.sanger.ac.uk/cosmic).

### Statistical Analyses

Statistical analyses were conducted using GraphPad Prism v7 (GraphPad Software, LaJolla, California, USA) or SPSS 21 (IBM, Armonk, NY, USA), and Student's t-tests were performed to compare treatment groups. p-values <0.05 were considered statistically significant.

## Results

All vehicle treated animals developed tumor. The time between tumor implantation and sacrifice in vehicle-treated animals was relatively similar in the three models, with a mean of 37, 38 and 44 days for PMCA-3, PMP-2 and PMCA-3, respectively.

**Figure 1:**
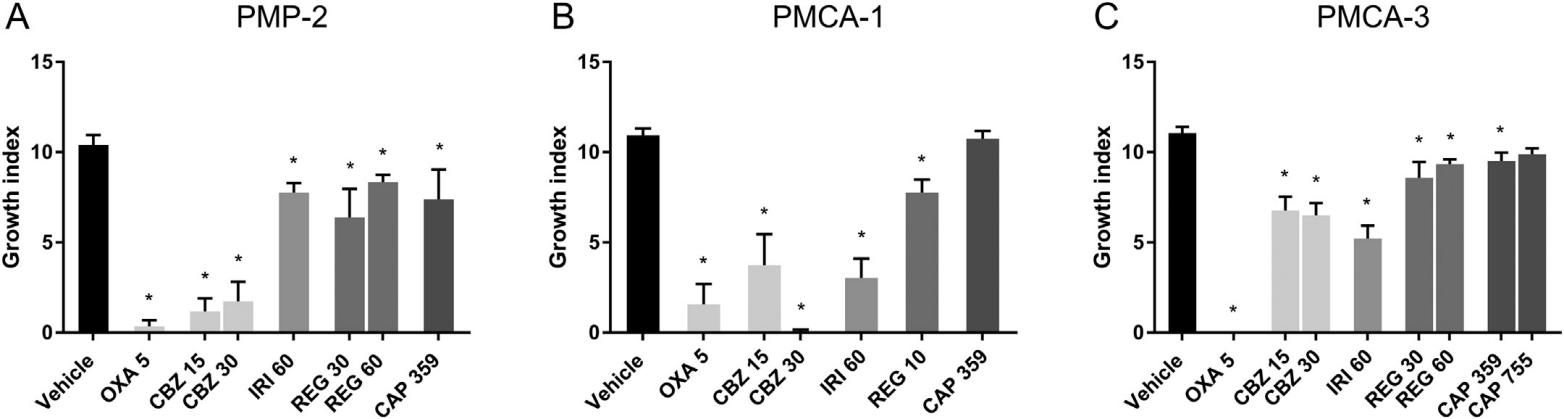
Growth index of (A) PMP-2, (B) PMCA-1 and (C) PMCA-3 treated with oxaliplatin (OXA), irinotecan (IRI), cabazitaxel (CBZ), regorafenib (REG) and capecitabine (CAP). Numbers on the X-axis indicate dose in mg/kg. * *P* < .05. Error bars indicate standard error of the mean (SEM).

In the PMP-2 model, OXA was the most efficacious drug, and 5 of 6 mice did not develop tumor growth, with a mean reduction of growth index of 97% compared to vehicle treatment. CBZ also had a strong growth inhibitory effect, with a mean reduction in growth index of 86% (*P* < .001), but no dose–response relationship was observed (Figure 1*A*). Of the 11 animals receiving CBZ, 6 did not develop tumor growth (Table 1). Moderate growth inhibition (20–29%) was observed with IRI, CAP and REG (*P* < .05). One animal in the CAP group did not develop tumor, otherwise all animals were sacrificed because of tumor growth.

**Table 1:** In vivo tumor growth in three models treated with vehicle, oxaliplatin, irinotecan, cabazitaxel, regorafenib or capecitabine with number of animals with tumor growth, survival, amount of tumor and number of animals excluded and for what reason

	Vehicle	Oxaliplatin (mg/kg	Irinotecan (mg/kg)	Cabazitaxel (mg/kg)		Regorafenib (mg/kg)			Capecitabine (mg/kg)	
		5	60	15	30	10	30	60	359	755
**PMP-2**										
Tumor growth/ total number of animals	12/12	1/6	6/6	3/6	2/5		5/5	6/6	5/6	
Survival (days, mean)	39	100	68	100	97		62	57	67	
Tumor (g), mean (SEM)	4.3 (0.4)	0.4 (0.4)	4.6 (0.3)	1.2 (0.7)	1.4 (0.9)		2.6 (0.9)	4.1 (0.8)	4.1 (0.8)	
# of mice excluded					1*		1***			
**PMCA-1**										
Tumor growth/ total number of animals	18/18	0/6	5/6	3/6	1/6	12/12			6/6	
Survival (days, mean)	44	84	96	91	103	63			51	
Tumor (g), mean (SEM)	5.3 (0.3)	0 (0)	2.6 (1.0)	2.3 (0.9)	0.1 (0.1)	4.3 (0.4)			5.70 (0.3)	
**PMCA-3**										
Tumor growth/ total number of animals	24/24	0/5	6/6	9/10	5/5		10/11	6/6	11/11	5/5
Survival (days, mean)	37	100	74	68	75		51	46	45	44
Tumor (g), mean (SEM)	4.7 (0.3)	0	2.8 (0.6)	3.6 (0.5)	4.0 (0.5)		3.6 (0.5)	3.9 (0.3)	4.0 (0.4)	4.2 (0.4)
# of mice excluded		1**			1**		1**		1**	1**

(SEM, Standard error of the mean)

* Wrong treatment given.

** Mucin not weighed.

*** No tumor growth, only ascites.

In the PMCA-1 model, OXA was again the most efficacious drug, and none of the 6 mice receiving the treatment developed tumor (Figure 1*B*). Two mice were euthanized after 32 and 74 days due to snout infection and edema respectively, but no tumor was detected in the abdomen at the time of sacrifice. CBZ also had a strong growth inhibitory effect with a trend towards a dose–response effect (*P* = .06), with 66% and 99% reduction of growth inhibition (*P* < .01) after administration of 15 and 30 mg/kg, respectively. Complete growth control was observed in 5/6 mice and 3/6 mice for 30 mg/kg and 15 mg/kg, respectively. IRI also had a strong growth inhibitory effect, with a reduction in growth index of 72% (*P* < .001). One of six mice treated with IRI did not develop tumor, while the remaining mice either lived until the end of the experiment or close to the end. For REG 10 mg/kg, all animals developed tumor, and a moderate growth inhibition was observed, with a reduction in growth index of 29% (P < .001) (Table 1). CAP was the least efficacious drug, with no difference in growth index compared to vehicle treatment (Figure 1*B*).

PMCA-3 was the least responsive model, and except for OXA, only moderate tumor growth inhibition was achieved (Figure 1*C*). None of the five mice treated with OXA developed tumor. IRI and CBZ were equally effective in inhibiting tumor growth, with a mean reduction of the growth index to 53% for IRI (*P* < .001) and 39 and 41% for mice treated with 15 and 30 mg/kg CBZ, respectively (P < .001). Moderate growth inhibition was also observed with REG, with a reduction in growth index of 22% (*P* = .003) and 15% (*P* = .02) after treatment with 30 and 60 mg/kg REG, respectively. One mouse treated with 30 mg/kg REG did not develop tumor, otherwise all animals were sacrificed due to tumor growth (Table 1). A small reduction of growth index was observed in mice treated with CAP with a 14% (*P* = .014) and 11% (*P* = .139) reduction in growth index after administration of 359 and 755 mg/kg, respectively (Figure 1*C*).

### Mutation Analyses

PMP-2 and PMCA-3 both had mutated *GNAS* (R201C)*,* while mutated *KRAS* (G12V and G12A) was present in PMP-2 and PMCA-1. In addition, mutations in *BRAF* (V600E) and *CTNNB1* (D32G) were detected in PMCA-3 and a *TP53* (R248Q) was detected in PMCA-1 (Table 2).

**Table 2:** Overview over genes mutated in the PMP-2, PMCA-1 and PMCA-3 models

	*KRAS*	*GNAS*	*BRAF*	*TP53*	*CTNNB1*
PMP-2	p.G12V	p.R201C	wt	wt	wt
PMCA-1	p.G12A	wt	wt	p.R248Q	wt
PMCA-3	wt	p.R201C	p.V600E	wt	p.D32G

## Discussion

Single i.p. injections of OXA 5 and 10 mg/kg were previously investigated in the same models with a modest inhibitory effect on tumor growth [12], and for this study the schedule was changed to include four weekly administrations of 5 mg/kg. The strong growth inhibitory effect observed was interesting, highlighting the importance of the treatment schedule. The results from the PRODIGE7 trial presented at ASCO in 2018 [19] suggested that adding OXA-based HIPEC to CRS in PM-CRC did not improve the survival compared to CRS alone, leading to questions regarding the benefit of HIPEC in the treatment of PM-CRC [19,20]. OXA exposure time has been shown to be associated with response in vitro, and the 30-minute OXA exposure in the PRODIGE7 trial may have been insufficient to cause effective tumor cell killing [20,21]. Our results suggest that repeated exposure might improve drug efficacy, and points to OXA having a strong direct anti-tumor effect in these models upon administration in the peritoneal cavity.

Single injections of CBZ efficaciously inhibited tumor growth, and at the highest doses exhibited similar effects as were previously observed with single i.p. injections of MMC [12]. CBZ is a member of the taxane family of microtubuli inhibitors, preventing cell division by stabilizing the microtubuli [22]. It is approved for treatment of hormone refractory prostate cancer following docetaxel-based treatment, but ongoing clinical trials are investigating CBZ in other cancer types as well [23]. In metastatic CRC (mCRC), a phase IIb trial was terminated because of lack of responses to intravenous administration in the first 10 included patients (NCT02204332). The strong growth inhibition observed in our experiments suggests a potential opportunity for CBZ in peritoneal disease, possibly administered i.p. Interestingly, i.p. injections of IRI also inhibited tumor growth in all models. High expression of topoisomerase 1, which is associated with response to IRI [24] was frequently detected in PM-CRC (54% in a cohort of 465 cases) and also in PMP (63% in a cohort of 43 cases) [25,26], providing a molecular rationale to expect efficacy of IRI in peritoneal disease. IRI is a component of standard-of-care systemic treatment for mCRC, but has not been extensively administrated in i.p. treatment protocols [5,27,28]. Experimental results have suggested favorable treatment responses and less toxicity with i.p. compared to intravenous injection of IRI, highlighting the potential for locoregional treatment [14]. When used in combination with OXA during HIPEC, no increase in survival was observed for patients receiving the combination compared to OXA alone [29].

Significant growth inhibition was observed upon oral administration of REG in all the investigated models, but with no clear dose response effects. REG is a multi-tyrosine kinase inhibitor targeting angiogenic, stromal and oncogenic receptor tyrosine kinases, and is approved for treatment of mCRC [30,31]. REG has a short half-life, thus the requirement for daily administration of the drug. Treatment was stopped after 4 weeks, and since it has previously been observed that REG will inhibit tumor growth only on treatment, complete responses would not be expected [31]. Prolongation of the treatment period would have been expected to lengthen the inhibition of tumor growth. A possible combination strategy for REG is together with IRI, which has previously been shown to result in increased progression-free survival in patients with mCRC [32]. Interestingly, very encouraging results have also been observed with REG in combination with nivolumab in microsatellite stable mCRC in the recently reported REGONIVO trial, suggesting a novel opportunity for systemic treatment in PM-CRC [33].

The fluoropyrimidine, CAP, was the least efficacious drug in our models, having either a weak or no inhibitory effect on tumor growth. Fluoropyrimidines are included as a backbone of mCRC treatment, typically in combination with OXA or IRI [34]. Intravenous administration of 5-fluorouracil is also by many centers included concomitantly with HIPEC in patients with PMP and PM-CRC in combination with MMC or OXA [35,36]. In line with our observations, fluoropyrimidine monotherapy is not extensively administered in this setting, and is not expected to be highly efficacious.

The growth inhibitory responses varied between the models, with PMP-2 and PMCA-1 being more responsive than the PMCA-3 model. The PMCA-3 model, being a high-grade mucinous cancer with signet ring cell differentiation, mimics an aggressive phenotype, which could contribute to explain the observed differences. *KRAS* and *GNAS* mutations were the most frequent mutations observed in our models, both being common mutations in PM-CRC and PMP, respectively [25,26], indicating that the PDX models are representative of the original disease and relevant models for investigating drug responses. None of the mutations detected in our models are predictive biomarkers for the drugs investigated in this study. *KRAS* and *GNAS* mutations are both associated with poor survival in patients with CRC and PMP [7,37,38], and treatments that exhibit efficacy in models with these genomic aberrations could potentially have an impact on a large patient group. Mutated *KRAS* has generally been considered to be undruggable, but efforts to develop drugs targeting *KRAS* have been extensive. Recently, a novel small molecule inhibitor of *KRAS* G12C (AMG 510) was shown to induce stable disease or partial responses in the majority of patients with advanced solid tumors receiving the treatment [39,40]. With *KRAS* mutations being prevalent in PM, efficacious targeting of mutated *KRAS* would represent an important therapeutic breakthrough for mutant PM cases.

In this study, OXA, CBZ and IRI administered by i.p. injection, all resulted in substantial growth inhibition, supporting the concept of locoregional administration of cytotoxic drugs in the peritoneal cavity. In contrast, the orally administered drugs, REG and CAP, were less efficacious, all though REG had an apparent inhibitory effect as long as it was being administered, and could be considered in combinatory protocols. There is a clear unmet need for new treatments for patients with PM-CRC and PMP, and based on the current work, OXA remains and CBZ emerges as particularly interesting candidates for further studies. We are currently investigating nanoparticle-based delivery of these drugs for i.p. administration.

## Acknowledgements

KGF and CL-A are postdoctoral fellows of the 10.13039/100008730Norwegian Cancer Society (Grant No. [197837]). This work was also supported by funding from the 10.13039/100001260National Organization for Rare Disorders (NORD) through Appendix Cancer Pseudomyxoma Peritonei (ACPMP) Research Foundation.

## Conflict of Interest

Bayer AG provided regorafenib and research support for the study.

## Author Contributions

**KGF**: Investigation, formal analysis, visualization, writing original draft. **CLA**: Formal analysis, investigation. **SW**: Investigation. **TWA**: Resources. **YM**: Resources. **KB**: Conceptualization. **AT**: Formal analysis, investigation. **KF**: Conceptualization, methodology, writing reviewing and editing, supervision, funding acquisition. All authors approved the manuscript.
